# Legacy effect of microplastics on plant–soil feedbacks

**DOI:** 10.3389/fpls.2022.965576

**Published:** 2022-08-08

**Authors:** Yudi M. Lozano, Matthias C. Rillig

**Affiliations:** ^1^Plant Ecology, Institute of Biology, Freie Universität Berlin, Berlin, Germany; ^2^Berlin-Brandenburg Institute of Advanced Biodiversity Research (BBIB), Berlin, Germany

**Keywords:** microplastic shape, plant–soil interactions, plant biomass, polymer type, root morphological traits, soil inocula

## Abstract

Microplastics affect plants and soil biota and the processes they drive. However, the legacy effect of microplastics on plant–soil feedbacks is still unknown. To address this, we used soil conditioned from a previous experiment, where *Daucus carota* grew with 12 different microplastic types (conditioning phase). Here, we extracted soil inoculum from those 12 soils and grew during 4 weeks a native *D. carota* and a range-expanding plant species *Calamagrostis epigejos* in soils amended with this inoculum (feedback phase). At harvest, plant biomass and root morphological traits were measured. Films led to positive feedback on shoot mass (higher mass with inoculum from soil conditioned with microplastics than with inoculum from control soil). Films may decrease soil water content in the conditioning phase, potentially reducing the abundance of harmful soil biota, which, with films also promoting mutualist abundance, microbial activity and carbon mineralization, would positively affect plant growth in the feedback phase. Foams and fragments caused positive feedback on shoot mass likely *via* positive effects on soil aeration in the conditioning phase, which could have increased mutualistic biota and soil enzymatic activity, promoting plant growth. By contrast, fibers caused negative feedback on root mass as this microplastic may have increased soil water content in the conditioning phase, promoting the abundance of soil pathogens with negative consequences for root mass. Microplastics had a legacy effect on root traits: *D. carota* had thicker roots probably for promoting mycorrhizal associations, while *C. epigejos* had reduced root diameter probably for diminishing pathogenic infection. Microplastic legacy on soil can be positive or negative depending on the plant species identity and may affect plant biomass primarily *via* root traits. This legacy may contribute to the competitive success of range-expanding species *via* positive effects on root mass (foams) and on shoot mass (PET films). Overall, microplastics depending on their shape and polymer type, affect plant–soil feedbacks.

## Introduction

Microplastics (<5 mm) are an important new global change factor with known effects on terrestrial ecosystems worldwide (Rillig, 2012; Xu et al., 2019). These particles may appear in different shapes (e.g., fibers, films, foams, fragments) and sizes, spanning a wide range of physical and chemical properties (Rochman et al., 2019; Helmberger et al., 2020). Microplastics (MPs) can pollute the soil through different pathways such as soil amendments, plastic mulching, irrigation, flooding, atmospheric input, littering, and street runoff (Rillig, 2012; Bläsing and Amelung, 2018; Xu et al., 2019; Brahney et al., 2020), causing recognized effects on soil properties and soil biota.

Microplastics as a function of their shape may affect soil aggregation, soil bulk density, nutrient retention, or soil pH (De Souza Machado et al., 2019; Zhao et al., 2021; Lozano et al., 2021a,b). In the shape of films, MPs may create additional channels for water movement, increasing the rate of soil evaporation (Wan et al., 2019); while by contrast, in the shape of fibers MPs may hold water in the soil for longer, enhancing soil water retention (De Souza Machado et al., 2019; Lozano et al., 2021b). In addition, microplastic foams or fragments may increase soil porosity and aeration (Carter and Gregorich, 2006; Ruser et al., 2008). All these microplastic effects on soil properties may in turn have consequences for soil biota (conditioned soil biota). Indeed, it is known that MPs as a function of their shape may affect soil microbial respiration, enzymatic activity, or soil microbial community composition (Fei et al., 2020; Zhao et al., 2021; Lozano et al., 2021b).

Microplastics made from different polymer types have different effects on soil properties and biota (Lozano et al., 2021b). In order to prolong plastic life and enhance polymer properties such as flexibility, durability, color, or resistance (Hahladakis et al., 2018; Waldman and Rillig, 2020), additives such as light and thermal stabilizers, UV absorbers, colored pigments, anti-fog substances, and antioxidants (Hahladakis et al., 2018) are used in plastic manufacture. Many of these additives may potentially leach into the soil with harmful consequences and toxic effects on soil biota. Negative effects of microplastic have been detected on nematode reproduction (Kim et al., 2020), earthworm performance (Huerta Lwanga et al., 2016), as well as on soil microorganisms. In fact, microplastics may cause a decline in soil bacterial diversity and richness (Huang et al., 2019; Fei et al., 2020), and also potentially cause negative effects on soil fungal communities (Kettner et al., 2017; Leifheit et al., 2021; Zhu et al., 2022).

Previous research shows that microplastics may promote shoot and root mass as a function of their shape and polymer type (Lozano et al., 2021b), responses that have been linked to microplastic effects on soil properties, as for example, the increase of soil macroporosity (Carter and Gregorich, 2006; Ruser et al., 2008), which facilitates root penetration and thus plant growth. By contrast, microplastics could also cause a reduction in plant growth if they, for instance, contain toxic substances (Van Kleunen et al., 2020). These positive or negative effects of microplastics on plant performance can also be explained by plant–soil feedbacks. That is, the microplastic legacy effect on soil biota and their subsequent consequences or feedback on plant performance (Bever et al., 1997; Van der Putten et al., 2016); a phenomenon that may occur if the microplastic is present in the soil or if it is removed (e.g., after being degraded or transported down in the soil profile). Microplastics in soil can feedback on plant species either positively, negatively, or neutrally (*sensu* Bever et al., 1997). Positive feedback (an increase in plant performance driven by microplastic legacy) or negative feedback (the opposite) can be linked with the accumulation of soil biota that can improve plant performance, such as beneficial rhizosphere bacteria, fixing nitrogen bacteria, mutualistic fungi (Bever et al., 1997), or with the accumulation of pathogens that can suppress plant growth, such as pathogenic bacteria and fungi or nematodes (Bever et al., 1997; Lithner et al., 2011; Van der Putten et al., 2016; Hahladakis et al., 2018; Bennett and Klironomos, 2019).

Microplastics’ legacy on soil would also be linked with the creation of “new habitat conditions” (novel environments) that may favor some plant species, as for instance, those of invasive character. Previous research has shown that microplastics may promote the growth of species of invasive character over other native species, as observed with the range-expanding species *Calamagrostis epigejos* (Lozano and Rillig, 2020), however, the mechanisms underlying this phenomenon are still unknown. One possibility is that the microplastic legacy on soil may positively feedback on range-expanding species while negatively on other native species. This, as mechanisms that promote plant invasiveness such as facilitation by soil biota or release of natural enemies (Daneshgar and Jose, 2009) would be potentially promoted by the microplastics legacy on soil, which in the end may help explain the competitive success of species of invasive character in novel environments.

The implications of the legacy effect of microplastics in soil have not yet been elucidated, despite interest in legacy effects of global change factors on terrestrial ecosystems (Meisner et al., 2013; Duell et al., 2019). Thus, we hypothesize that the legacy effect of microplastics on soil would affect the magnitude and direction of the feedback on plants depending on the microplastic shape and polymer type with which the soil was conditioned, as well as on the plant trait studied. We included root traits since in addition to plant biomass, root morphological traits are key indicators of plant–soil feedback responses to global change factors (Lozano et al., 2022). Likewise, our study aims to understand whether the legacy effect of microplastics on soil may contribute to the competitive success of range-expanding species. To do this, a controlled experiment was established where *Daucus carota* (a dryland native plant species) and *C. epigejos* (a dryland range-expanding species) grew with inoculum extracted from soil previously conditioned by different microplastic shapes and polymer types. We expect feedback responses of the plant species, depending on the microplastic type with which the soil was conditioned, and an increase in the performance of the range-expanding species due to the legacy effect of microplastics on soil.

## Materials and methods

### Soil conditioning phase (previous experiment)

In a previous experiment (Lozano et al., 2021b), sandy loam soil was conditioned for 8 weeks with 12 different microplastic types. That is, soil was mixed at a concentration of 0.4% w/w (0.4 g of microplastic for each 100 g of dry soil) with microplastics that had different shapes (i.e., fibers, films, foams, or fragments), each one made of three of the following polymer types: polyester made of at least 80% of polyethylene terephthalate (PES), polyamide (PA), polypropylene (PP), low-density polyethylene (LDPE), called polyethylene from now on, polyethylene terephthalate (PET), polyurethane (PU), polystyrene (PS), and polycarbonate (PC; Table 1 illustrates which polymer was used for each microplastic shape). Thus, the experimental design included 4 microplastic shapes × 3 polymer types. Control pots without microplastics were also included. Soil was incubated for 2 weeks, and then, for each of the 12 microplastic types, 7 replicates were established where a single seedling of *D. carota* grew in each pot during 4 weeks. At harvest, soil free of roots was air-dried for 2 weeks and immediately, sampled for using in this experiment (the feedback phase). See additional details of the conditioning phase in Lozano et al. (2021b).

**Table 1:** List of the plastics (shapes and polymer types) used in the conditioned phase, general characteristics of the plastic and the source of them are mentioned.

Shape	Polymer	Characteristics	Source
Fiber	Polyester (PES)	PES is made of at least 80% of polyethylene terephthalate. PET’s major uses are textiles, strapping, films and engineering moldings. This resin is commonly used in beverage bottles	Rope Paraloc Mamutec polyester white, item number, 8442172, Hornbach.de
	Polyamide (PA)	Fibers of polyamide are widely used in textiles, automotive industry and sportswear due to their high durability and strength	Connex, item number 10010166, Hornbach.de
	Polypropylene (PP)	PP present a high chemical resistance, is strong, and has a high melting point which make it widely used for hot-fill liquids. Several household items are made of this polymer	Rope Paraloc Mamutec polypropylene orange, item number, 8442182, Hornbach.de
Film	Low density Polyethylene (LDPE)	LDPE is mostly used in film applications due to its toughness, flexibility and relative transparency. LDPE is also used to manufacture flexible lids, bottles and packaging	Silo film black, folien-bernhardt.de
	Polyethylene terephthalate (PET)	See comments above on Polyester (PES)	Company: Toppits/product: oven bag
	Polypropylene (PP)	See comments above	Company: STYLEX/product: transparent folders
Foam	Low-density Polyethylene (LDPE)	See comments above	Black low-density closed cell ETHAFOAM polyethylene foam; alibaba.com
	Polystyrene (PS)	PS can be rigid or foamed. It has a relatively low melting point. Typical applications include protective packaging, food service packaging, bottles, and food containers	EPS70 Insulation Packing Board SLABS, Wellpack Europe
	Polyurethane (PU)	PU can be thermosetting or thermoplastic, rigid and hard or flexible and soft. Products include mattresses, adhesives, coatings, sealants	Gray foam sheet, item number, 3838930, Hornbach.de
Fragment	Polycarbonate (PC)	The main advantage of polycarbonate is unbeatable strength combined with light weight. Plastic used for a variety of applications, from bulletproof windows to compact disks (CDs)	CD-R Verbatim
	Polyethylene terephthalate (PET)	See comments above on Polyester (PES)	Vio Still, item number 41005958, vio.de
	Polypropylene (PP)	See comments above	Black plastic pots, treppens.de

Information about plastic characteristics were extracted from plastic resins code table (LEED’s) and the polymer properties database (polymerdatabase.com).

### Soil inoculum preparation

We prepared the soil inoculum to be use in the feedback phase, following Rodríguez-Echeverría et al. (2013) and Lozano et al. (2017). That is, we took 75 g of soil from each replicate of the conditioning phase and stirred for 5 min in 150 ml of distilled, autoclaved water in a 1:2 (v:v) ratio. Then, the soil mixture was passed through a 0.5 mm sieve to remove soil particles, allowing fungal spores, hyphae, soil bacteria and microfauna to pass through (Van de Voorde et al., 2012). The soil extracts collected were used to inoculate the pots according to the experimental design. One soil extract (i.e., inoculum) per replicate was prepared. For the control replicates, the same procedure was followed but using our sandy loam soil neither sterilized nor conditioned with microplastics. This inoculum preparation procedure reduced any relative potential differential input of nutrients or microplastic residues with inoculation (Rodríguez-Echeverría et al., 2013). Small microplastics or nanoplastics could be present in the soil inoculum.

### Plant species selection

For the feedback phase, we selected *D. carota* and *C. epigejos* as phytometers, because these species exhibit clear responses to the addition of microplastics in the soil (Lozano and Rillig, 2020; Lozano et al., 2021b). *Daucus carota* is a native biennial herbaceous plant typical of grassland ecosystems (Federal Agency for Nature Conservation, 2019) and constitutes a “conspecific” feedback, since it grows in the soil that it had conditioned; while *C. epigejos* is a native species of range-expanding character (Těšitel et al., 2017), which appears to perform better (higher biomass) than other natives within a grassland community when microplastics are added into the soil (Lozano and Rillig, 2020) and constitutes, for this experiment, a “heterospecific” feedback, since it grows in the soil conditioned by *D. carota*. Both plant species are native in Central Europe. Seeds of these plant species were obtained from commercial suppliers in the region (Rieger-Hofmann GmbH, Blaufelden, and Jelitto Staudensamen GmbH, Schwarmstedt, Germany, respectively), surface sterilized with 4% sodium hypochlorite for 5 min and 75% ethanol for 2 min, thoroughly rinsed with sterile water and germinated on sterile sand. Then, 3 days after germination, seedlings of similar size were used in this experiment.

### Feedback phase

In November 2019, we collected and sieved (4 mm mesh size) sandy loamy soil (Albic Luvisol; 0.07% N, 0.77% C, pH 6.66) from Dedelow, Brandenburg, Germany (53° 37′N, 13° 77′) where our plant species naturally grow in a diverse dry grassland, and as it was the same soil type used in the conditioning phase. Soil was autoclaved three times for 20 min at 120°C and then used as sterile substrate in pots (pot of 6 cm diameter, 25 cm height, 500 ml, 400 g of capacity). Soil sterilization could have increased nutrient concentration in soil due to the decomposition of soil organisms, phenomenon that especially occurs in nutrient rich soils (Powlson and Jenkinson, 1976), which is not the case here (Albic Luvisol). Nonetheless, the unlikely increase in nutrients does not imply a bias in our experimental design as all the substrate soil was subjected to the same treatment before being placed into the pots. After this, the soil was inoculated with inoculum from an independent soil replicate and 10 days later 98 seedlings of each of the two plant species were transplanted as single individuals into each pot. Thus, our experimental design included 2 plant species × 12 different soil inocula (from each of the 12 microplastic types: 4 shapes × 3 polymer types) × 7 replicates = 168 pots. Fourteen additional control pots were established per plant species. Plants in the feedback phase grew for 6 weeks. All pots were watered twice per week with 70 ml of water to keep water holding capacity ~60%. Plants were grown in a glasshouse chamber with a daylight period set at 12 h, 50 klx, and a temperature regime at 22/18°C day/night with relative humidity of ~40%. None of the plants died during the experiment. Pots were randomly distributed in the chamber and their position shifted twice to homogenize environmental conditions during the experiment.

### Measurements

At the end of the experiment, roots were carefully removed from the soil and gently washed in order to measure morphological traits in fine roots (i.e., <2 mm in diameter which included mostly first to third order roots). We measured length, surface area, volume and root average diameter on a fresh sample using the WinRhizoTM scanner-based system (v.2007; Regent Instruments Inc., Quebec, Canada). We calculated different root morphological traits: specific root surface area (SRSA; cm^2^ mg^−1^), specific root length (SRL; cm mg^−1^), root average diameter (RAD; mm) and root tissue density (RTD; root dry weight per volume mg cm^−3^). Shoot and root mass were measured after drying samples at 70°C for 48 h.

### Statistical analyses

In order to test whether microplastics in soil have feedback effects on plant performance, we evaluated the effects of soil conditioned by microplastics on shoot and root masses and on root traits, through linear models and multiple comparisons (“multcomp” R package). Therefore, inocula from soil conditioned with microplastics having different shapes and polymer types (microplastic type) were considered as fixed factors. Residuals were checked to follow assumptions of normality and homogeneity and when necessary, we implemented the function “varIdent” to account for heterogeneity. After that, we implemented the function “glht” and the “Dunnett” test from the “multcomp” R package (Hothorn et al., 2008; Bretz et al., 2011), in order to compare the effect of the inoculum from soil conditioned by microplastics with the control (inoculum from soil without being conditioned by microplastics). In addition, effect sizes were estimated to show the variability in the response of our variables (plant biomass and root traits), by comparing the effect of each soil inoculum conditioned by microplastics with the effect of soil inoculum from the control pots by using a bootstrap-coupled estimation “dabestr” R package (Ho et al., 2019). Positive effects indicated that shoot and root mass were greater with the inoculum from soil conditioned by microplastics than with inoculum from soil not conditioned by microplastics (positive feedback). Negative effects indicate the opposite (negative feedback), while neutral effects indicate a similar response among treatments (neutral feedback). Root trait responses were analyzed in a similar way. Positive numbers indicate a higher value of the trait with inoculum from soil conditioned by microplastics than with inoculum from soil not conditioned by MPs, while negative numbers indicate the opposite. All data were analyzed for each plant species separately using R v.3.5.3. (R Core Team, 2019).

## Results

Microplastics in soil had a legacy effect on plant species, which depended on the microplastic shape and polymer type with which the soil was previously conditioned, and on the plant trait studied.

### Microplastic feedback effects on the native *Daucus carota*: Changes in biomass and root traits are evident

**Figure 1:**
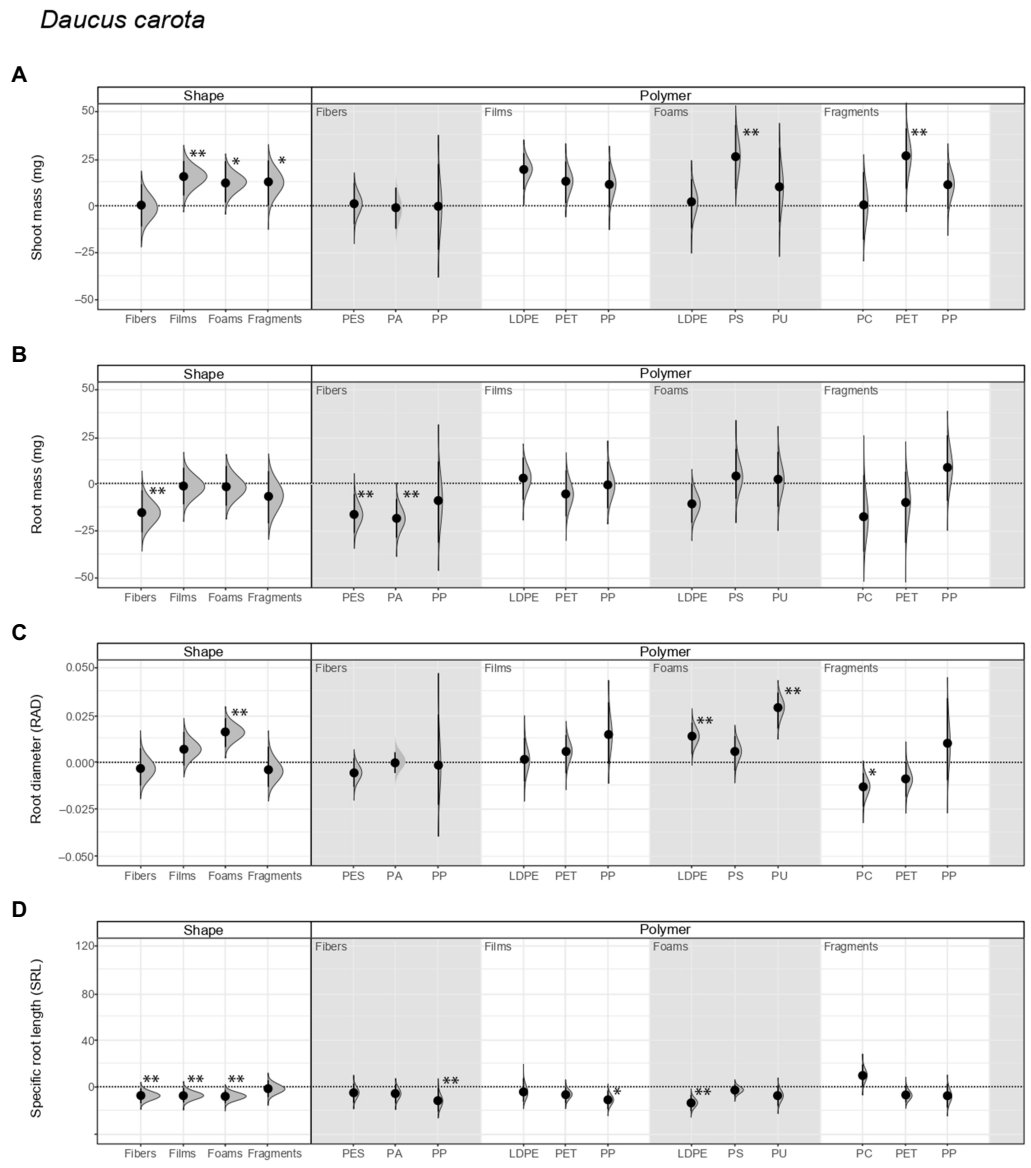
Legacy effect of microplastic shape and polymer type on **(A)** shoot mass, **(B)** root mass, **(C)** root average diameter (RAD), and **(D)** specific root length (SRL) of *Daucus carota*. Effect sizes and their variance are displayed as means and 95% confidence intervals. Horizontal dotted line indicates the mean value of the control (soil conditioned without microplastics). Polymers: PES, polyester; PA, polyamide; PP, polypropylene; LDPE, low-density polyethylene; PET, polyethylene terephthalate; PS, polystyrene; PU, polyurethane; PC, polycarbonate. Strong and moderate evidence was established at 0.05 (**) and 0.1 (*), respectively (Tables 2, 3). *n* = 7 for soil conditioned with microplastics, *n* = 14 for control samples.

We found that shoot mass increased by ~20% with inoculum from soil conditioned with films, ~17% with foams, and ~17% with fragments, in comparison to the control with inoculum not conditioned by microplastics (Figure 1A; Supplementary Figure S1; Table 2). Regarding polymer type, shoot mass increased by ~35% and ~36% with inoculum from soil conditioned with PS foams and PET fragments, respectively (Figure 1A; Table 3). By contrast, root mass decreased in average by ~22% with inoculum from soil conditioned by fibers. Regarding polymer type, it decreased by 25% and 28% with inoculum from soil conditioned by PES and PA fibers (Figure 1B; Tables 2, 3; see Supplementary Table S1 for absolute values used to calculate the percentage changes between treatments and the control).

**Table 2:** Legacy effect of microplastic shape on plant mass and root traits.

Linear model	df	Shoot mass		Root mass		RAD		SRL		RTD		SRSA	
		Daucus	Calamagrostis	Daucus	Calamagrostis	Daucus	Calamagrostis	Daucus	Calamagrostis	Daucus	Calamagrostis	Daucus	Calamagrostis
Shape	4	**4.04 (0.004)**	*2.27 (0.06)*	**2.59 (0.04)**	**7.23 (<0.01)**	**5.82 (<0.01)**	**3.12 (0.02)**	**2.65 (0.04)**	**2.88 (0.02)**	**4.08 (<0.01)**	**4.95 (0.01)**	*2.34 (0.06)*	**2.65 (0.04)**
Shape-control> = 0	Dunnett test: *z* and (*p* value)												
Fibers		−0.06 (0.83)	−1.00 (0.97)	**−2.59 (0.03)**	−1.77 (0.18)	−0.58 (0.96)	−1.38 (0.18)	**−2.31 (0.03)**	−0.15 (0.83)	**3.56 (<0.01)**	*2.07 (0.06)*	**−2.78 (<0.01)**	−0.75 (0.84)
Films		**3.14 (0.003)**	1.29 (0.24)	−0.30 (0.99)	−0.32 (0.98)	1.55 (0.37)	**−2.13 (0.04)**	**−2.32 (0.03)**	−0.11 (0.81)	**2.28 (0.04)**	**2.52 (0.02)**	**−2.16 (0.05)**	−0.85 (0.78)
Foams		*2.01 (0.07)*	1.23 (0.25)	−0.33 (0.99)	*2.04 (0.10)*	**3.97 (<0.01)**	**−2.44 (0.02)**	**−2.70 (0.01)**	**2.52 (0.01)**	1.72 (0.14)	−1.64 (0.99)	*−2.01 (0.07)*	**2.41 (0.05)**
Fragments		*2.08 (0.06)*	0.73 (0.46)	−0.93 (0.73)	−0.12 (1.00)	−0.74 (0.89)	**−3.32 (0.01)**	−0.48 (0.61)	1.20 (0.26)	*2.11 (0.06)*	1.46 (0.20)	−0.95 (0.40)	0.26 (0.99)

Results of linear model (*F* and *p* value) and multiple comparisons by using the Dunnett test: *z* and (*p* value). Values in bold signify a strong effect (*p* < 0.05) and, in italic a moderate effect (*p* < 0.1) of the treatment on the dependent variable.

**Table 3:** Legacy effect of microplastic type effect on plant mass and root traits.

Linear model		Shoot mass		Root mass		RAD		SRL		RTD		SRSA	
	df	Daucus	Calamagrostis	Daucus	Calamagrostis	Daucus	Calamagrostis	Daucus	Calamagrostis	Daucus	Calamagrostis	Daucus	Calamagrostis
Legacy of microplastic type	12	**2.19 (0.01)**	**3.48 (<0.01)**	**3.44 (<0.01)**	**10.98 (<0.01)**	**6.28 (<0.01)**	**2.75 (<0.01)**	**2.56 (<0.01)**	**3.63 (<0.01)**	**2.93 (<0.01)**	**4.55 (<0.01)**	**2.55 (<0.01)**	**3.45 (<0.01)**
MPs-control >= 0	Dunnett’s test: *z* and (*p* value)												
Fibers	PES	0.06 (1.00)	−2.00 (0.31)	**−3.13 (0.01)**	**−3.89 (<0.01)**	−1.07 (0.97)	−1.49 (0.30)	−1.22 (0.55)	−0.09 (1.00)	1.62 (0.41)	1.18 (0.92)	1.62 (0.41)	1.18 (0.92)
	PA	−0.17 (1.00)	−0.85 (0.98)	**−3.44 (<0.01)**	*−2.52 (0.08)*	−0.38 (1.00)	−1.54 (0.28)	−1.08 (0.62)	−1.19 (0.86)	1.36 (0.58)	*2.73 (0.06)*	1.36 (0.58)	*2.73 (0.06)*
	PP	−0.03 (1.00)	0.06 (1.00)	−0.77 (0.99)	0.29 (1.00)	−0.11 (1.00)	−0.63 (0.71)	**−2.56 (0.05)**	0.60 (0.99)	**3.84 (<0.01)**	0.81 (0.99)	**3.84 (<0.01)**	0.81 (0.99)
Films	LDPE	2.26 (0.21)	0.22 (1.00)	0.4 (0.99)	−1.26 (0.78)	0.21 (1.00)	**−4.41 (<0.01)**	−0.93 (0.70)	0.80 (0.98)	1.02 (0.77)	2.36 (0.16)	1.02 (0.77)	2.36 (0.16)
	PET	1.53 (0.71)	**2.76 (0.05)**	−0.88 (0.98)	0.65 (0.99)	1.04 (0.97)	−0.26 (0.84)	−1.43 (0.43)	−0.98 (0.95)	1.25 (0.65)	1.33 (0.86)	1.25 (0.65)	1.33 (0.86)
	PP	1.39 (0.85)	0.24 (1.00)	−0.19 (1.00)	−0.52 (0.99)	1.61 (0.70)	−1.85 (0.17)	*−2.36 (0.08)*	−0.38 (0.99)	*2.42 (0.08)*	1.66 (0.62)	*2.42 (0.08)*	1.66 (0.62)
Foams	LDPE	0.27 (1.00)	1.04 (0.94)	−1.93 (0.38)	1.31 (0.75)	**3.04 (0.02)**	**−2.57 (0.03)**	**−2.92 (0.01)**	1.91 (0.35)	*2.51 (0.06)*	−1.79 (0.52)	*2.51 (0.06)*	−1.79 (0.52)
	PS	**3.06 (0.02)**	1.27 (0.83)	0.50 (0.99)	*2.50 (0.09)*	1.14 (0.95)	−1.64 (0.24)	−0.61 (0.83)	−0.21 (1.00)	0.02 (0.99)	1.07 (0.96)	0.02 (0.99)	1.07 (0.96)
	PU	1.19 (0.92)	0.63 (0.99)	0.22 (1.00)	1.38 (0.69)	**5.63 (<0.01)**	**−2.48 (0.04)**	−1.63 (0.33)	**3.59 (<0.01)**	0.28 (0.98)	**−3.05 (0.02)**	0.28 (0.98)	**−3.05 (0.02)**
Fragments	PC	0.07 (1.00)	1.43 (0.73)	−1.60 (0.63)	0.92 (0.95)	*−2.71 (0.07)*	**−2.67 (0.02)**	2.04 (1.00)	−0.12 (1.00)	−0.27 (0.99)	2.21 (0.23)	−0.27 (0.99)	2.21 (0.23)
	PET	**3.11 (0.02)**	−0.32 (1.00)	−0.99 (0.97)	−2.09 (0.23)	−1.76 (0.57)	**−2.67 (0.02)**	−1.51 (0.39)	1.41 (0.71)	*2.47 (0.07)*	0.25 (1.00)	*2.47 (0.07)*	0.25 (1.00)
	PP	1.32 (0.85)	0.29 (1.00)	0.85 (0.99)	0.32 (1.00)	0.84 (0.99)	**−3.17 (<0.01)**	−1.70 (0.30)	1.48 (0.66)	2.16 (0.15)	0.67 (0.99)	2.16 (0.15)	0.67 (0.99)

(*F* and *p* value) and multiple comparisons by using the Dunnett test: *z* and (*p* value). PP, Polypropylene; PES, polyester; PA, polyamide; PE, polyethylene; PET, polyethylene terephthalate; PU, polyurethane; PS, polystyrene; PC, polycarbonate. Values in bold evidence a strong effect (*p* < 0.05), and in italic a moderate effect (*p* < 0.1) of the treatment on the dependent variable.

*Daucus* root morphological traits were also influenced by the legacy effect of microplastics in soil. That is, root diameter (RAD) increased by ~9% with inoculum from soil conditioned by foams. Of these, it increased by ~8% and ~16% with inoculum from soil conditioned by LDPE and PU foams, respectively. Likewise, it decreased by ~7% with inoculum from soil conditioned by PC fragments (Figure 1C; Tables 2, 3). By contrast, specific root length (SRL) decreased with inoculum from soil conditioned by most of the microplastic shapes. SRL decreased by ~22%, ~21%, and ~23% with inoculum from soil conditioned by fibers, films, and foams, respectively. Of these, SRL decreased by ~34%, ~21%, and ~19% with inoculum from soil conditioned with PP fibers, PP films, and LDPE foams, respectively (Figure 1D; Tables 2, 3; Supplementary Table S1).

Other root morphological traits were also affected by the legacy of microplastics in soil. Root tissue density (RTD) increased by ~52% with inoculum from soil conditioned with fibers, ~23% with films, and ~21% with fragments (Supplementary Figure S2A; Table 2; Supplementary Table S1). Regarding polymer type, RTD increased by ~91%, ~31%, ~57%, and ~39% with inoculum from soil conditioned with PP fibers, PP films, LDPE foams, and PET fragments, respectively. Similar to specific root length (SRL), specific root surface area (SRSA) decreased by ~24%, ~19%, and ~17% with inoculum from soil conditioned by fibers, films, and foams (Supplementary Figure S2B; Table 2; Supplementary Table S1). Of these, SRSA decreased by ~69, 38% and ~54, and 30% with inoculum from soil conditioned with PP fibers, PP films, LDPE foams, and PET fragments, respectively.

### Microplastic feedback effects on the native range-expanding *Calamagrostis epigejos*: Effects on shoot mass are practically negligible, although effects on root mass are evident

**Figure 2:**
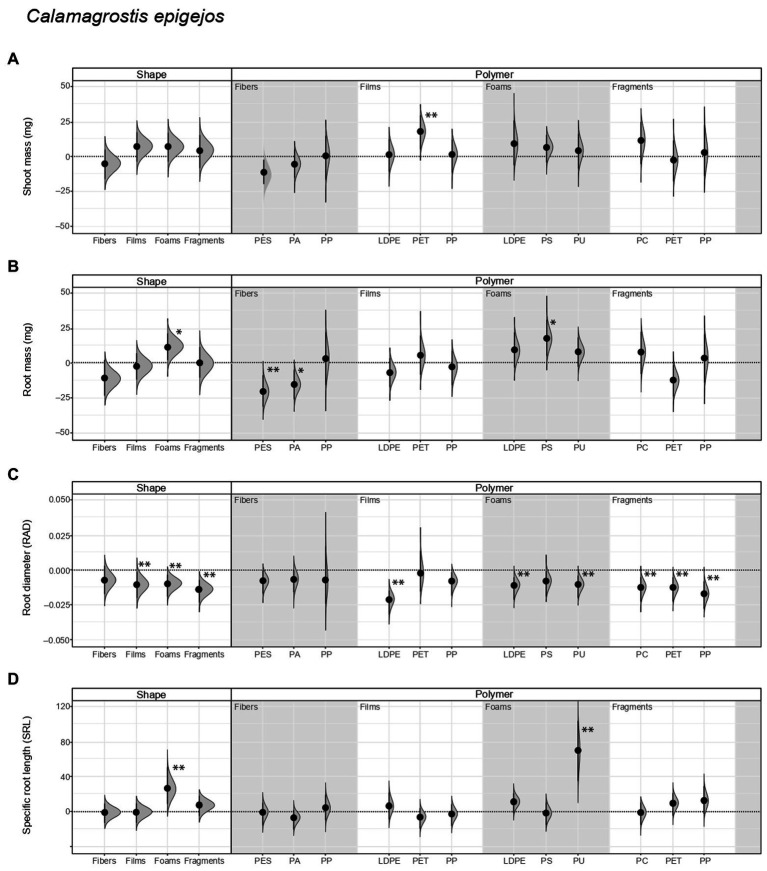
Legacy effect of microplastic shape and polymer type on **(A)** shoot mass, **(B)** root mass, **(C)** root average diameter (RAD) and **(D)** specific root length (SRL) of *Calamagrostis epigejos*. Effect sizes and their variance are displayed as means and 95% confidence intervals. Horizontal dotted line indicates the mean value of the control (soil conditioned without microplastics). Polymers: PES, polyester; PA, polyamide; PP, polypropylene; LDPE, low-density polyethylene; PET, polyethylene terephthalate; PS, polystyrene; PU, polyurethane; PC, polycarbonate. Strong and moderate evidence was established at 0.05 (**) and 0.1 (*), respectively (Tables 2, 3). *n* = 7 for soil conditioned with microplastics, *n* = 14 for control samples.

We did not find evidence that shoot mass of *Calamagrostis* was affected by soil inoculum as a function of having been conditioned by microplastics of different shapes. Nonetheless, regarding polymer type, shoot mass increased by ~32% with inoculum from soil conditioned with PET films (Figure 2A; Tables 2, 3; Supplementary Table S1). Root mass increased by ~21% with inoculum from soil conditioned by foams, in comparison to the control not conditioned by microplastics (Figure 2B; Table 2). Regarding polymer type, it increased by ~33% with inoculum from soil conditioned with PS foams while it decreased by ~40% and ~29% with inoculum from soil conditioned by PES and PA fibers (Figure 2B; Table 3; Supplementary Table S1).

With respect to root morphological traits, we found that root diameter (RAD) decreased by ~8% with inoculum from soil conditioned by films, ~7% by foams and 11% by fragments (Figure 2C; Table 2; Supplementary Table S1). Regarding polymer type, RAD decreased by ~17%, ~9% and ~9% with inoculum from soil conditioned by LDPE films, LDPE, and PU foams, respectively; and by ~10%, ~10%, and ~14% with inoculum from soil conditioned by PC, PET, and PP fragments, respectively (Figure 2C; Table 3; Supplementary Table S1). By contrast, specific root length (SRL) increased by ~50% with inoculum from soil conditioned with foams, in comparison to the control not conditioned by microplastics. Of these, SRL increased by ~132% with inoculum from soil conditioned with PU foams (Figure 2D; Tables 2, 3; Supplementary Table S1).

Likewise, root tissue density (RTD) increased by ~15% and ~19% with inoculum from soil conditioned by fibers and films, respectively, in comparison to the control not conditioned by microplastics. Regarding polymer type, RTD increased by ~22% with inoculum from soil conditioned with PA fibers, while by contrast, it decreased by ~37% with inoculum from soil conditioned by PU foams (Supplementary Figure S3A; Tables 2, 3; Supplementary Table S1). On the other hand, specific root surface area (SRSA) increased by ~44% with inoculum from soil conditioned by foams. Of these, SRSA increased by ~123% with inoculum from soil conditioned by PU foams (Supplementary Figure S3B; Tables 2, 3; Supplementary Table S1).

### Comparative effect of the legacy of microplastics on native and range-expanding species

Our results showed that the legacy of microplastics in soil affect the performance of the native *D. carota* and the range-expanding species *C. epigejos* (Figures 1, 2). When comparing the size of the effect of each species minus the control, we observed that in terms of shoot mass, films and foams were more positive for *Daucus* than for *Calamagrostis* (films had a mean difference of 14.4 mg for *Daucus* and 6.83 mg for *Calamagrostis*; while foams had a mean difference of 12.7 mg and 6.5 mg, respectively, Table 4). By contrast, PET film was more positive for *Calamagrostis* than for *Daucus* (17.6 mg and 12.9 mg, respectively, Table 4). In terms of root mass, foams were more positive for *Calamagrostis* than for *Daucus* (11, −1.8, respectively), being PS the foam that most promoted *Calamagrostis* over *Daucus* (17, 3.59, respectively).

**Table 4:** Unpaired mean difference (mg) of the legacy effect of each microplastic shape and polymer type minus control.

Shoot mass					Root mass			
Microplastic	Daucus		Calamagrostis		Daucus		Calamagrostis	
	Mean	95 CI	Mean	95 CI	Mean	95 CI	Mean	95 CI
Fibers	−0.39	−11.6; 10.4	−5.29	−16; 4.65	**−15.2**	−25.5; −3.58	**−10.9**	−22; 1.26
Films	14.4	4.82; 22.8	6.83	−3.79; 16.8	−1.53	−10.8; 8.22	−1.79	−11.9; 9.06
Foams	12.7	0.609; 25.1	6.55	−3.73; 16.5	**−1.81**	−11.5; 9.08	**11**	1.08; 21.5
Fragments	12.7	0.554; 24.1	3.9	−8.25; 14.9	**−6.61**	−20.8; 6.45	**−0.76**	−12.2; 10.9
Fiber (PES)	0.54	−13.4; 12.3	−10.7	−21.6; −1.64	−17.2	−27.3; −7.05	−20.7	−31.1; −10.5
Fiber (PA)	−1.44	−12.4; 8.08	−5.68	−17.5; 6.72	**−19.1**	−28.7; −8.41	**−15**	−25.6; −3.59
Fiber (PP)	**−0.26**	−22.7; 21.9	**0.53**	−16.2; 14.6	**−9.23**	−31.1; 11.2	**2.9**	−15.8; 21.3
Film (LDPE)	19.1	8.71; 27.6	1.36	10.6; 12.3	2.47	−8.82; 13.2	−6.87	−17.2; 3.11
Film (PET)	**12.9**	1.55; 23.2	**17.6**	5.13; 28.9	**−5.79**	−17.5; 6.52	**4.86**	−8.47; 19
Film (PP)	11.3	−0.734; 23.2	1.49	−11.2; 11.9	−1.29	−13.2; 11.1	−3.36	−15.8; 8.12
Foam (LDPE)	**2.26**	−11.5; 14.1	**9.08**	−6.14; 25.4	**−10.8**	−20.8; −0.437	**8.54**	−3.41; 21.2
Foam (PS)	25.8	8.9; 42.2	6.44	−4.09; 15.2	**3.59**	−8.26; 17.7	**17**	4.69; 30.1
Foam (PU)	**2.1**	−8.36; 30.4	**4.15**	−8.48; 15.9	**1.8**	−12.4; 16.2	**7.57**	−2.85; 17.6
Fragment (PC)	**0.64**	−17.5; 17.8	**11.3**	−5.29; 24.3	**−17.6**	−35.8; 3.99	**7.27**	−8.06; 21.6
Fragment (PET)	26.2	9.07; 40.5	−2.45	−17.3; 11.3	−10.2	−31.3; 5.81	−12.6	−23.8; −1.82
Fragment (PP)	11.2	−1.48; 22.5	2.86	−13.5; 22.1	8	−9.37; 24.8	3	−13.8; 19.9

Data Analysis with Bootstrap Estimation using Dabestr. Values correspond to those used in Figures 1, 2. Confidence intervals (CI) at 95%. In bold when the mean difference was positive and higher for *Calamagrostis* than for *Daucus* meaning that the legacy effect of microplastics promoted the growth of the range-expanding *Calamagrostis* over the native species *Daucus.* (see Tables 2, 3).

## Discussion

**Figure 3:**
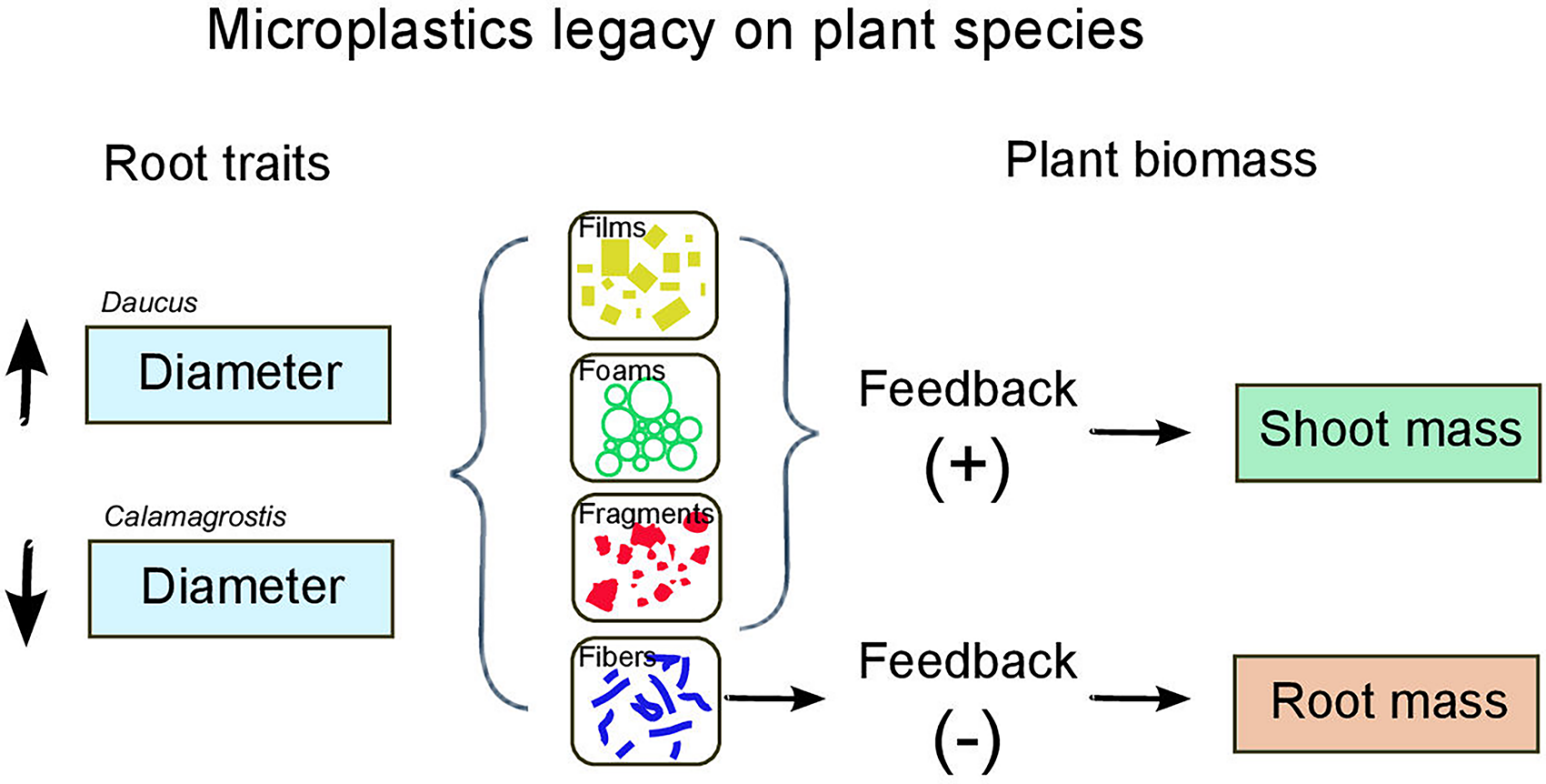
Summary of microplastics legacy on plant species: effects on plant biomass and root traits.

Our results showed that microplastics had a legacy effect on soil with consequences for plant biomass and root morphological traits depending on plant species identity (Figure 3). We found that microplastics can cause a positive or negative feedback on plants depending on the microplastic shape and polymer type that previously had conditioned the soil.

### Microplastic films led to a positive feedback on shoot mass of *Daucus carota*

Microplastic films led to a positive feedback on shoot mass (higher mas with inoculum from soil conditioned by MPs than in the control), which may be linked with microplastic films increasing in the conditioning phase, soil enzymatic activities such as urease or catalase (Huang et al., 2019) as well as the abundance of nitrogen fixers bacteria (Fei et al., 2020). The priming effect of the carbon in microplastics (the addition of carbon to the soil due to MPs) may have positively affected the mineralization of native soil organic C in that conditioning phase (Rillig et al., 2021), helping to explain the subsequent positive effect in the feedback phase. Likewise, microplastic films in soil may have affected soil water status, as they increase the rate of soil evaporation (Wan et al., 2019). Previous research has found that fungal pathogens associated with *D. carota* decrease in abundance when soil water is reduced (Lozano et al., 2021c). A similar relationship between pathogen abundance and water reduction has been observed (Buscardo et al., 2021). Thus, the presence of microplastics films in soil in the conditioning phase, may have reduced pathogen abundance and other harmful soil biota *via* effects on soil water status (something to be tested), which, added to the likely promotion of mutualists abundance, microbial activity and carbon mineralization (Huang et al., 2019; Fei et al., 2020; Rillig et al., 2021), may help explain the positive effect on shoot mass of *D. carota* caused by the legacy of microplastic films (positive feedback). Nonetheless, our results also found that PET films led to a positive feedback on shoot mass of the range-expanding species *Calamagrostis*.

### Microplastic foams and fragments led to a positive feedback on shoot mass of *Daucus carota*

The positive feedback on shoot mass caused by microplastic foams and fragments may be linked with their positive effects on soil aeration in the conditioning phase (Lozano et al., 2021b), and as consequence, on the soil microbial activity and soil biota present in the feedback phase (Bronick and Lal, 2005). Previous research has observed that soil aeration induced by microplastics increases enzymatic activities, bacterial diversity, and the relative abundance of beneficial soil bacteria associated to nitrification and nitrogen fixation (Qian et al., 2022). Likewise, it has been shown that microplastic foams mixed with soil increase enzymatic activities such as cellobiosidase, β-D-glucosidase, and N-acetylβ-glucosaminidase (Zhao et al., 2021). Thus, microplastic foams and fragments in soil may have promoted beneficial soil biota in the conditioning phase *via* effects on soil aeration, which as consequence may help explain the positive effect that inoculum from soil conditioned with these microplastics have on shoot mass of *D. carota* (positive feedback).

The polymer type of which microplastic foams and fragments were made also played a role in plant–soil feedback. We observed that inocula from soil conditioned by PS foams and PET fragments were those that cause a positive effect on shoot mass in the feedback phase. In that sense, previous results show that PS foams in the conditioning phase increase soil enzyme activity such as for β-glucosidase and cellobiosidase (Awet et al., 2018), reason why subsequent positive feedback on shoot mass can be expected. However, PS or PET can also negatively affect other enzymes in the conditioning phase, as these MPs are made of monomers that can be potentially hazardous for the environment (Lithner et al., 2011).

### Microplastic fibers led to a negative feedback on root mass of both plant species

Contrary to microplastic films, foams or fragments, we found that microplastic fibers had a negative feedback on root mass. Microplastic fibers in the conditioning phase could help hold water for longer increasing soil water content (De Souza Machado et al., 2019; Lozano et al., 2021b), a soil water status that appears to increase the abundance of fungal pathogens associated with *D. carota* (Lozano et al., 2021c). As a consequence, in the feedback phase, plants might have had a decreased root mass as due to pathogenic infection.

### Microplastics and their legacy effect on root morphological traits

Our results showed that the legacy effect of most microplastic shapes caused a decrease in root fineness of *D. carota*. From the root economic spectrum perspective, SRL and SRSA negatively correlate with root diameter (Wright et al., 2004; Reich, 2014), a situation that was most evident with inoculum from soil conditioned by microplastic foams. As mentioned, MPs foams may promote in the conditioning phase, the abundance of mutualistic soil biota, a microbial group which is highly linked with root diameter (Buscardo et al., 2021). Therefore, in the feedback phase, *D. carota* could have developed thicker roots with low fineness, perhaps in order to promote mycorrhizal fungi associations (Brundrett, 2002; Weemstra et al., 2016; Kong et al., 2017; Lozano et al., 2020), which support a faster nutrient acquisition (Wahl and Ryser, 2000; Withington et al., 2006), helping explain the positive feedback in terms of shoot mass for *D. carota*. However, the legacy effect of microplastics on *C. epigejos* was different than that on *D. carota*. We found that overall, inoculum from soil conditioned by microplastics decreased root diameter of *C. epigejos* (higher root fineness), which evidence a different strategy that promote a positive feedback in terms of shoot mass for *Calamagrostis*. That is because the fine roots helps to establish positive associations with saprotrophs communities (Semchenko et al., 2018; Lozano et al., 2021c), which promote carbon mineralization in soil with positive effects on plant performance.

### Microplastic legacy in soil affects plant species depending on their identity: They may promote the growth of range-expanding over native species

We obtained strong evidence that microplastics in soil had a legacy effect on shoot mass of *D. carota* that, with most microplastics, was greater than the legacy effect on shoot mass of *Calamagrostis*. However, microplastics as PET films promoted the shoot mass of the range-expanding *C. epigejos* over the native *D. carota*. Likewise, the legacy of microplastic foams in the soil promoted the root mass of the range-expanding over the native species. Although this did not translate to shoot mass during the experiment, this stronger positive effect of PET films and foams on root mass suggest that the range-expanding species may have a competitive advantage over the native species in terms of resource uptake and formation of symbiotic associations, which in the end may favor their establishment in the field. Our results showed that microplastics legacy on soil may contribute to the competitive success of this range-expanding species.

Likewise, we found that root morphological traits are strongly affected by the microplastics legacy on soil and that depending on the plant species a different root trait is affected. The legacy effect of microplastics on plants are first experienced by the roots, as those are in direct contact with the soil, which was observed for both species, then, the effect may be transferred to plant biomass, depending on the plant species. For example, unlike *D. carota*, which was affected in its root traits and plant biomass by microplastics legacy, *C. epigejos* was affected in its root traits, but this effect did not extend to plant biomass, which shows that microplastics effects on plants are species-specific, as is the case with other global change factors (Lozano et al., 2020).

Microplastics in soil left a legacy that could promote the growth of species of invasive character. However more research is needed in this regard. For example, the success of many species of invasive character is linked to a rapid germination (Lozano et al., 2019), a key plant life stage that can be influenced by the legacy of microplastics in soil. Likewise, as microplastics interplay with drought affecting soil ecosystem functionality (Lozano et al., 2021a), how the legacy of microplastics in soil may act in combination not only with drought but with other global change factors needs to be addressed. Finally, future research on this topic should include a variety of plant species, as we observed that the responses to the legacy of microplastic in soil were species-specific. Experiments using single species, in intra or interspecific interaction, and in a community, would help us understand microplastics effects on plant community assembly and may contribute to validate our findings about microplastics legacy promoting species of invasive character over native species.

Overall, our results showed that microplastics have a legacy effect on plant biomass and root morphological traits which can be positive (higher values with inoculum from soil conditioned by MPs than with inoculum from control soils) or negative (the opposite) as a function of microplastic shape and polymer type. Certainly, the positive feedback does not mean a desirable effect but simply an increase in plant biomass and alterations in root morphological traits. Indeed, the presence of an effect, even a positive one, implies an alteration of the natural state. Our study provides novel insights into the effects of microplastics on terrestrial systems highlighting their key role in plant–soil feedbacks.

## Data availability statement

Data that support the findings of this study are available in figshare as: Lozano, Yudi M.; Rillig, Matthias (2022): PSF_MPS_data.csv. figshare. Dataset. https://doi.org/10.6084/m9.figshare.20238957.v1

## Author contributions

YL conceived the ideas, designed the methodology with input from MR, established and maintained the experiment in the greenhouse, analyzed the data, and wrote the first draft. MR contributed to the draft with his comments and editions. All authors contributed to the article and approved the submitted version.

## Funding

The work was funded by the German Federal Ministry of Education and Research (BMBF) within the collaborative Project “Bridging in Biodiversity Science (BIBS)” (funding number 01LC1501A). MR additionally acknowledges support from the EU grants MINAGRIS and PAPILLONS, and from an ERC Advanced Grant (694368).

## Conflict of interest

The authors declare that the research was conducted in the absence of any commercial or financial relationships that could be construed as a potential conflict of interest.

## Publisher’s note

All claims expressed in this article are solely those of the authors and do not necessarily represent those of their affiliated organizations, or those of the publisher, the editors and the reviewers. Any product that may be evaluated in this article, or claim that may be made by its manufacturer, is not guaranteed or endorsed by the publisher.

## References

[ref1] Awet T. T. Kohl Y. Meier F. Straskraba S. Grün A.-L. Ruf T.. (2018). Effects of polystyrene nanoparticles on the microbiota and functional diversity of enzymes in soil. Environ. Sci. Eur. 30:11. doi: 10.1186/s12302-018-0140-6, PMID: 29963347 PMC5937892

[ref2] Bennett J. A. Klironomos J. (2019). Mechanisms of plant–soil feedback: interactions among biotic and abiotic drivers. New Phytol. 222, 91–96. doi: 10.1111/nph.15603, PMID: 30451287

[ref3] Bever J. D. Westover K. M. Antonovics J. (1997). Incorporating the soil community into plant population dynamics: the utility of the feedback approach. J. Ecol. 85, 561–573. doi: 10.2307/2960528

[ref4] Bläsing M. Amelung W. (2018). Plastics in soil: analytical methods and possible sources. Sci. Total Environ. 612, 422–435. doi: 10.1016/j.scitotenv.2017.08.086, PMID: 28863373

[ref5] Brahney J. Hallerud M. Heim E. Hahnenberger M. Sukumaran S. (2020). Plastic rain in protected areas of the United States. Science 368, 1257–1260. doi: 10.1126/science.aaz5819, PMID: 32527833

[ref6] Bretz F. Hothorn T. Westfall P. (2011). Multiple Comparisons Using R. New York, NY: Chapman and Hall/CRC, 205.

[ref7] Bronick C. J. Lal R. (2005). Soil structure and management: a review. Geoderma 124, 3–22. doi: 10.1016/j.geoderma.2004.03.005

[ref8] Brundrett M. C. (2002). Coevolution of roots and Mycorrhizas of land Plants. New Phytol. 154, 275–304. doi: 10.1046/j.1469-8137.2002.00397.x, PMID: 33873429

[ref9] Buscardo E. Souza R. C. Meir P. Geml J. Schmidt S. K. da Costa A. C. L.. (2021). Effects of natural and experimental drought on soil Fungi and biogeochemistry in an Amazon rain Forest. Commun Earth Environ. 2:55. doi: 10.1038/s43247-021-00124-8

[ref10] Carter M. R. Gregorich E. G. (2006). Soil Sampling and Methods of Analysis. Boca Raton, FL: CRC Press.

[ref11] Daneshgar P. Jose S. (2009). “Mechanisms of plant invasion: a review,” in Invasive Plants and Forest Ecosystems. eds. Kohli R. K. Jose S. Singh H. P. Batish D. R. (New York, NY: CRC press), 437.

[ref12] De Souza Machado A. A. Lau C. W. Kloas W. Bergmann J. Bachelier J. B. Faltin E.. (2019). Microplastics can change soil properties and affect plant performance. Environ. Sci. Technol. 53, 6044–6052. doi: 10.1021/acs.est.9b01339, PMID: 31021077

[ref13] Duell E. B. Zaiger K. Bever J. D. Wilson G. W. T. (2019). Climate affects plant-soil feedback of native and invasive grasses: negative feedbacks in stable but not in variable environments. Front. Ecol. Evol. 7:419. doi: 10.3389/fevo.2019.00419

[ref14] Federal Agency for Nature Conservation (2019). Floraweb [online]. Available at: http://floraweb.de/index.html (Accessed May 14, 2019).

[ref15] Fei Y. Huang S. Zhang H. Tong Y. Wen D. Xia X.. (2020). Response of soil enzyme activities and bacterial communities to the accumulation of microplastics in an acid cropped soil. Sci. Total Environ. 707:135634. doi: 10.1016/j.scitotenv.2019.135634, PMID: 31761364

[ref16] Hahladakis J. N. Velis C. A. Weber R. Iacovidou E. Purnell P. (2018). An overview of chemical additives present in plastics: migration, release, fate and environmental impact during their use, disposal and recycling. J Hazard. Mater 344, 179–199. doi: 10.1016/j.jhazmat.2017.10.014., PMID: 29035713

[ref17] Helmberger M. S. Tiemann L. K. Grieshop M. J. (2020). Towards an ecology of soil microplastics. Funct. Ecol. 34, 550–560. doi: 10.1111/1365-2435.13495

[ref18] Ho J. Tumkaya T. Aryal S. Choi H. Claridge-Chang A. (2019). Moving beyond P values: data analysis with estimation graphics. Nat. Methods 16, 565–566. doi: 10.1038/s41592-019-0470-3, PMID: 31217592

[ref19] Hothorn T. Bretz F. Westfall P. (2008). Simultaneous inference in general parametric models. Biom. J. 50, 346–363. doi: 10.1002/bimj.200810425, PMID: 18481363

[ref20] Huang Y. Zhao Y. Wang J. Zhang M. Jia W. Qin X. (2019). LDPE microplastic films alter microbial community composition and enzymatic activities in soil. Environ. Pollut. 254:112983. doi: 10.1016/j.envpol.2019.112983, PMID: 31394342

[ref21] Huerta Lwanga E. Gertsen H. Gooren H. Peters P. Salánki T. van der Ploeg M.. (2016). Microplastics in the terrestrial ecosystem: implications for *Lumbricus Terrestris* (Oligochaeta, Lumbricidae). Environ. Sci. Technol. 50, 2685–2691. doi: 10.1021/acs.est.5b05478, PMID: 26852875

[ref22] Kettner M. T. Rojas-Jimenez K. Oberbeckmann S. Labrenz M. Grossart H.-P. (2017). Microplastics alter composition of fungal communities in aquatic ecosystems: fungal communities on microplastics. Environ. Microbiol. 19, 4447–4459. doi: 10.1111/1462-2920.13891, PMID: 28805294

[ref23] Kim S. W. Waldman W. R. Kim T.-Y. Rillig M. C. (2020). Effects of different microplastics on nematodes in the soil environment: tracking the extractable additives using an ecotoxicological approach. Environ. Sci. Technol. 54, 13868–13878. doi: 10.1021/acs.est.0c04641, PMID: 33052669 PMC7643727

[ref24] Kong D. Wang J. Zeng H. Liu M. Miao Y. Wu H.. (2017). The nutrient absorption–transportation hypothesis: optimizing structural traits in absorptive roots. New Phytol. 213, 1569–1572. doi: 10.1111/nph.14344., PMID: 27859373

[ref25] Leifheit E. F. Lehmann A. Rillig M. C. (2021). Potential effects of microplastic on arbuscular mycorrhizal fungi. Front. Plant Sci. 12:626709. doi: 10.3389/fpls.2021.626709, PMID: 33597964 PMC7882630

[ref26] Lithner D. Larsson Å. Dave G. (2011). Environmental and health Hazard ranking and assessment of plastic polymers based on chemical composition. Sci. Total Environ. 409, 3309–3324. doi: 10.1016/j.scitotenv.2011.04.038, PMID: 21663944

[ref27] Lozano Y. M. Aguilar-Trigueros C. A. Flaig I. C. Rillig M. C. (2020). Root trait responses to drought are more heterogeneous than leaf trait responses. Funct. Ecol. 34, 2224–2235. doi: 10.1111/1365-2435.13656

[ref28] Lozano Y. M. Aguilar-Trigueros C. A. Onandia G. Maaß S. Zhao T. Rillig M. C. (2021a). Effects of microplastics and drought on soil ecosystem functions and multifunctionality. J. Appl. Ecol. 58, 988–996. doi: 10.1111/1365-2664.13839

[ref29] Lozano Y. M. Aguilar-Trigueros C. A. Ospina J. M. Rillig M. C. (2022). Drought legacy effects on root morphological traits and plant biomass *via* soil biota feedback. New Phytol. doi: 10.1111/nph.18327, PMID: 35719096

[ref30] Lozano Y. M. Aguilar-Trigueros C. A. Roy J. Rillig M. C. (2021c). Drought induces shifts in soil fungal communities that can be linked to root traits across 24 plant species. New Phytol. 232, 1917–1929. doi: 10.1111/nph.17707, PMID: 34480754

[ref31] Lozano Y. M. Armas C. Hortal S. Casanoves F. Pugnaire F. I. (2017). Disentangling above-and below-ground facilitation drivers in arid environments: the role of soil microorganisms, soil properties and microhabitat. New Phytol. 216, 1236–1246. doi: 10.1111/nph.14499, PMID: 28262957

[ref32] Lozano Y. M. Hortal S. Armas C. Pugnaire F. I. (2019). Soil micro-organisms and competitive ability of a tussock grass species in a dry ecosystem. J. Ecol. 107, 1215–1225. doi: 10.1111/1365-2745.13104

[ref33] Lozano Y. M. Lehnert T. Linck L. T. Lehmann A. Rillig M. C. (2021b). Microplastic shape, polymer type, and concentration affect soil properties and plant biomass. Front. Plant Sci. 12:616645. doi: 10.3389/fpls.2021.616645, PMID: 33664758 PMC7920964

[ref34] Lozano Y. M. Rillig M. C. (2020). Effects of microplastic fibers and drought on plant communities. Environ. Sci. Technol. 54, 6166–6173. doi: 10.1021/acs.est.0c01051, PMID: 32289223 PMC7241422

[ref35] Meisner A. De Deyn G. B. de Boer W. van der Putten W. H. (2013). Soil biotic legacy effects of extreme weather events influence plant invasiveness. PNAS 110, 9835–9838. doi: 10.1073/pnas.1300922110, PMID: 23716656 PMC3683719

[ref36] Powlson D. S. Jenkinson D. S. (1976). The effects of biocidal treatments on metabolism in soil—II. Gamma irradiation, autoclaving, air-drying and fumigation. Soil Biol. Biochem. 8, 179–188. doi: 10.1016/0038-0717(76)90002-X

[ref37] Qian Z. Zhuang S. Gao J. Tang L. Harindintwali J. D. Wang F. (2022). Aeration increases soil bacterial diversity and nutrient transformation under mulching-induced hypoxic conditions. Sci. Total Environ. 817:153017. doi: 10.1016/j.scitotenv.2022.153017, PMID: 35026241

[ref38] R Core Team (2019). R: A Language and Environment for Statistical Computing. Vienna: R Foundation for Statistical Computing.

[ref39] Reich P. B. (2014). The world-wide ‘fast-slow’ plant economics spectrum: a traits manifesto. J. Ecol. 102, 275–301. doi: 10.1111/1365-2745.12211

[ref40] Rillig M. C. (2012). Microplastic in terrestrial ecosystems and the soil? Environ. Sci. Technol. 46, 6453–6454. doi: 10.1021/es302011r 22676039

[ref41] Rillig M. C. Leifheit E. Lehmann J. (2021). Microplastic effects on carbon cycling processes in soils. PLoS Biol. 19:e3001130. doi: 10.1371/journal.pbio.3001130, PMID: 33784293 PMC8009438

[ref42] Rochman C. M. Brookson C. Bikker J. Djuric N. Earn A. Bucci K.. (2019). Rethinking microplastics as a diverse contaminant suite. Environ. Toxicol. Chem. 38, 703–711. doi: 10.1002/etc.4371, PMID: 30909321

[ref43] Rodríguez-Echeverría S. Armas C. Pistón N. Hortal S. Pugnaire F. I. (2013). A role for below-ground biota in plant-plant facilitation. J. Ecol. 101, 1420–1428. doi: 10.1111/1365-2745.12159

[ref44] Ruser R. Sehy U. Weber A. Guster R. Munch J. C. (2008). “Main driving variables and effect of soil management on climate or ecosystem-relevant trace gas fluxes from fields of the FAM,” in Perspectives for Agroecosystem Management. eds. Schroeder P. Pfadenhauer J. Munch J. C. (Amsterdam: Elsevier).

[ref001] Semchenko M. Leff J. W. Lozano Y. M. Saar S. Davison J. Wilkinson A.. (2018). Fungal diversity regulates plant-soil feedbacks in temperate grassland. Sci. Adv. 4:eaau4578. doi: 10.1126/sciadv.aau4578 30498781 PMC6261650

[ref45] Těšitel J. Mládek J. Horník J. Těšitelová T. Adamec V. Tichý L. (2017). Suppressing competitive dominants and community restoration with native parasitic Plants using the Hemiparasitic *Rhinanthus Alectorolophus* and the dominant grass *Calamagrostis Epigejos*. J. Appl. Ecol. 54, 1487–1495. doi: 10.1111/1365-2664.12889.

[ref46] Van de Voorde T. F. J. Van der Putten W. H. Bezemer T. M. (2012). Soil inoculation method determines the strength of plant–soil interactions. Soil Biol. Biochem. 55, 1–6. doi: 10.1016/j.soilbio.2012.05.020.

[ref47] Van der Putten W. H. Bradford M. A. Pernilla Brinkman E. Voorde T. F. J. Veen G. F. (2016). Where, when and how plant–soil feedback matters in a changing world. Funct. Ecol. 30, 1109–1121. doi: 10.1111/1365-2435.12657

[ref48] Van Kleunen M. Brumer A. Gutbrod L. Zhang Z. (2020). A microplastic used as infill material in artificial sport turfs reduces plant growth. Plants People Planet 2, 157–166. doi: 10.1002/ppp3.10071

[ref49] Wahl S. Ryser P. (2000). Root tissue structure is linked to ecological strategies of grasses. New Phytol. 148, 459–471. doi: 10.1046/j.1469-8137.2000.00775.x, PMID: 33863023

[ref50] Waldman W. R. Rillig M. C. (2020). Microplastic research should embrace the complexity of secondary particles. Environ. Sci. Technol. 54, 7751–7753. doi: 10.1021/acs.est.0c02194, PMID: 32559095 PMC8220496

[ref51] Wan Y. Wu C. Xue Q. Hui X. (2019). Effects of plastic contamination on water evaporation and desiccation cracking in soil. Sci. Total Environ. 654, 576–582. doi: 10.1016/j.scitotenv.2018.11.123, PMID: 30447596

[ref52] Weemstra M. Mommer L. Visser E. J. W. Ruijven J. Kuyper T. W. Mohren G. M. J.. (2016). Towards a multidimensional root trait framework: a tree root review. New Phytol. 211, 1159–1169. doi: 10.1111/nph.14003, PMID: 27174359

[ref53] Withington J. M. Reich P. B. Oleksyn J. Eissenstat D. M. (2006). Comparison of sructure and life span in roots and leaves among temperate trees. Ecol. Monogr. 76, 381–397. doi: 10.1890/0012-9615(2006)076[0381:COSALS]2.0.CO,2

[ref54] Wright I. J. Reich P. B. Westoby M. Ackerly D. D. Baruch Z. Bongers F.. (2004). The worldwide leaf economics Spectrum. Nature 428, 821–827. doi: 10.1038/nature02403, PMID: 15103368

[ref55] Xu B. Liu F. Cryder Z. Huang D. Lu Z. He Y.. (2019). Microplastics in the soil environment: occurrence, risks, interactions and fate: a review. Crit. Rev. Environ. Sci. Technol. 50, 2175–2222. doi: 10.1080/10643389.2019.1694822

[ref57] Zhao T. Lozano Y. M. Rillig M. C. (2021). Microplastics increase soil PH and decrease microbial activities as a function of microplastic shape, polymer type, and exposure time. Front. Environ. Sci. 9:675803. doi: 10.3389/fenvs.2021.675803

[ref58] Zhu D. Ma J. Li G. Rillig M. C. Zhu Y.-G. (2022). Soil Plastispheres as hotspots of antibiotic resistance genes and potential pathogens. ISME J. 16, 521–532. doi: 10.1038/s41396-021-01103-9, PMID: 34455424 PMC8776808

