# Supplementary material for: Legacy effect of microplastics on plant–soil feedbacks
Source: Front Plant Sci. 2022 Aug 8;13:965576. doi: 10.3389/fpls.2022.965576 (PMC9393594; doi:10.3389/fpls.2022.965576)

**Supporting information**

**Table S1.** Average values used to calculate the percentage changes between treatments and the control. Units: Shoot mass (mg), root mass (mg), root averge diameter (RAD, mm), specific root length (SRL, cm mg^-1^), root tissue density (RTD, mg cm^-3^), Specific root surface area (SRSA, cm^2^ mg^-1^). Polymer types: Polyamide (PA); Polyester (PES); Polypropylene (PP); Low-Density Polyethylene (LDPE); Polyethylene terephthalate (PET); Polystyrene (PS); Polyurethane (PU); Polycarbonate (PC).

| Treatment | Shoot mass | | Root mass | | RAD | | SRL | | RTD | | SRSA | |
| --- | --- | --- | --- | --- | --- | --- | --- | --- | --- | --- | --- | --- |
|  | ***Daucus*** | ***Calamagrostis*** | ***Daucus*** | ***Calamagrostis*** | ***Daucus*** | ***Calamagrostis*** | ***Daucus*** | ***Calamagrostis*** | ***Daucus*** | ***Calamagrostis*** | ***Daucus*** | ***Calamagrostis*** |
| Control | 73.07 | 55.44 | 67.62 | 51.6 | 0.17 | 0.12 | 31.25 | 46.84 | 148.11 | 193.89 | 1.66 | 1.76 |
| Fibers | 72.68 | 50.15 | 52.45 | 40.68 | 0.16 | 0.12 | 24.45 | 46.04 | 225.59 | 222.52 | 1.26 | 1.65 |
| Films | 87.5 | 62.26 | 66.09 | 49.81 | 0.17 | 0.11 | 24.66 | 46.25 | 181.94 | 230 | 1.36 | 1.63 |
| Foams | 85.77 | 61.99 | 65.82 | 62.64 | 0.19 | 0.11 | 24.04 | 70.39 | 178.49 | 169.01 | 1.39 | 2.54 |
| Fragments | 85.75 | 59.34 | 61.02 | 50.83 | 0.17 | 0.11 | 29.62 | 53.21 | 178.66 | 212.32 | 1.52 | 1.8 |

| Treatment |  | Shoot mass | | Root mass | | RAD | | SRL | | RTD | | SRSA | |
| --- | --- | --- | --- | --- | --- | --- | --- | --- | --- | --- | --- | --- | --- |
|  |  | ***Daucus*** | ***Calamagrostis*** | ***Daucus*** | ***Calamagrostis*** | ***Daucus*** | ***Calamagrostis*** | ***Daucus*** | ***Calamagrostis*** | ***Daucus*** | ***Calamagrostis*** | ***Daucus*** | ***Calamagrostis*** |
| Control | Control | 73.07 | 55.44 | 67.63 | 51.6 | 0.17 | 0.12 | 31.26 | 46.84 | 148.12 | 193.89 | 1.67 | 1.76 |
| Fibers | PES | 73.61 | 44.71 | 50.43 | 30.89 | 0.17 | 0.12 | 26.14 | 46.29 | 208.19 | 215.7 | 1.37 | 1.67 |
|  | PA | 71.63 | 49.76 | 48.53 | 36.65 | 0.17 | 0.12 | 26.72 | 40.79 | 185.17 | 237.51 | 1.43 | 1.48 |
|  | PP | 72.81 | 55.97 | 58.4 | 54.5 | 0.17 | 0.12 | 20.51 | 51.05 | 283.43 | 214.35 | 0.99 | 1.8 |
| Films | LDPE | 92.14 | 56.8 | 70.1 | 44.73 | 0.17 | 0.1 | 27.36 | 52.72 | 179.57 | 235.8 | 1.5 | 1.69 |
|  | PET | 86 | 73.06 | 61.84 | 56.46 | 0.18 | 0.12 | 25.27 | 41.41 | 172.47 | 230.76 | 1.39 | 1.59 |
|  | PP | 84.36 | 56.93 | 66.34 | 48.24 | 0.19 | 0.12 | 21.37 | 44.6 | 193.79 | 223.44 | 1.2 | 1.6 |
| Foams | LDPE | 75.33 | 64.51 | 56.8 | 60.14 | 0.19 | 0.11 | 19.02 | 56.91 | 232.27 | 172.82 | 1.08 | 2.04 |
|  | PS | 98.87 | 61.87 | 71.22 | 68.61 | 0.18 | 0.12 | 28.69 | 45.54 | 148.42 | 212.12 | 1.59 | 1.65 |
|  | PU | 83.13 | 59.58 | 69.43 | 59.17 | 0.2 | 0.11 | 24.42 | 108.73 | 154.78 | 122.09 | 1.5 | 3.91 |
| Fragments | PC | 73.72 | 66.73 | 50.02 | 58.87 | 0.16 | 0.11 | 39.8 | 46.1 | 142.83 | 233.63 | 1.97 | 1.59 |
|  | PET | 99.3 | 52.98 | 57.42 | 39.03 | 0.16 | 0.11 | 24.94 | 55.39 | 206.5 | 197.29 | 1.29 | 1.91 |
|  | PP | 84.23 | 58.3 | 75.63 | 54.6 | 0.18 | 0.11 | 24.12 | 58.16 | 186.64 | 206.03 | 1.29 | 1.91 |

**Figure S1**. Legacy effect of microplastics of differente shapes and polymer types on shoot and root mass of *Calamagrostis epigejos* and *Daucus carota.* Shapes: fiber, film, foam and fragment. Polymer types: PES (polyester), PA (polyamide), PP (polypropylene), LDPE (low-density polyethylene), PET (polyethylene terephthalate), PS (polystyrene), PU (polyurethane), and PC (polycarbonate). n=7 for soil previously conditioned with microplastics, n=14 for control samples (not conditioned by microplastics).


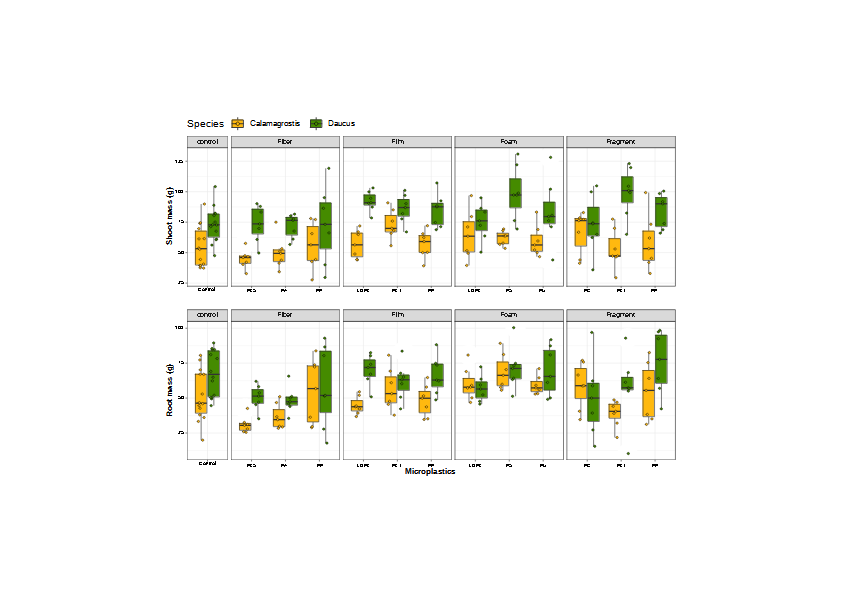


**Figure S2.** Legacy effect of microplastic shape and polymer type on (A) root tissue density (RTD) and (B) specific root surface area (SRSA) of *Daucus carota*. Effect sizes and their variance are displayed as means and 95% confidence intervals. Horizontal dotted line indicates the mean value of the control (soil conditioned without microplastics). Polymers: PES (polyester), PA (polyamide), PP (polypropylene), LDPE (low-density polyethylene), PET (polyethylene terephthalate), PS (polystyrene), PU (polyurethane), and PC (polycarbonate). Strong and moderate evidence was established at 0.05 (**) and 0.1 (*), respectively (Tables 2, 3). n=7 for soil conditioned with microplastics, n=14 for control samples.


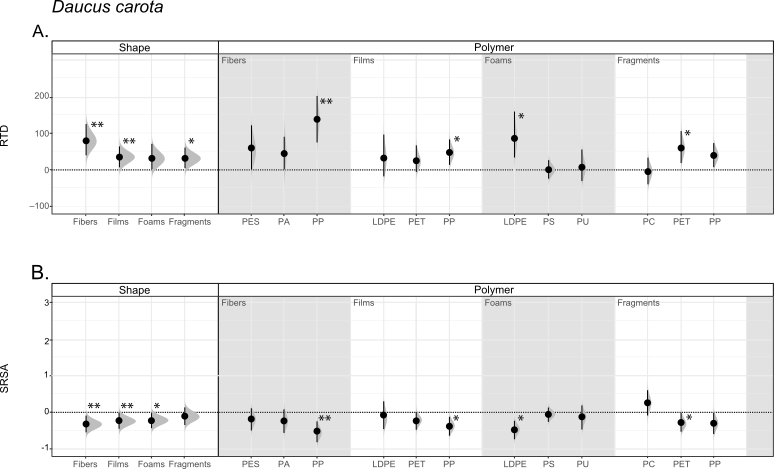


**Figure S3.** Legacy effect of microplastic shape and polymer type on (A) root tissue density (RTD) and (B) specific root surface area (SRSA) of *Calamagrostis epigejos*. Effect sizes and their variance are displayed as means and 95% confidence intervals. Horizontal dotted line indicates the mean value of the control (soil conditioned without microplastics). Polymers: PES (polyester), PA (polyamide), PP (polypropylene), LDPE (low-density polyethylene), PET (polyethylene terephthalate), PS (polystyrene), PU (polyurethane), and PC (polycarbonate). Strong and moderate evidence was established at 0.05 (**) and 0.1 (*), respectively (Tables 2, 3). n=7 for soil conditioned with microplastics, n=14 for control samples.


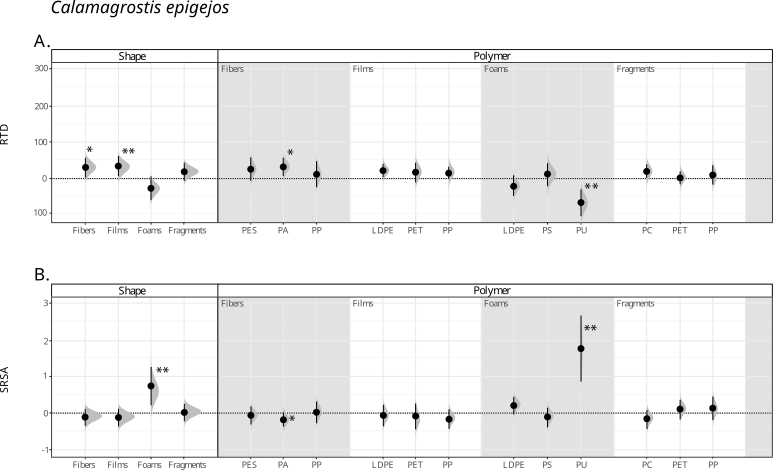

Supplement: Supplementary file 1 [file Data_Sheet_1.docx]
